# Occupational Injuries on Thoroughbred Horse Farms: A Description of Latino and Non-Latino Workers’ Experiences

**DOI:** 10.3390/ijerph10126500

**Published:** 2013-11-29

**Authors:** Jennifer E. Swanberg, Jessica M. Clouser, Susan C. Westneat, Mary W. Marsh, Deborah B. Reed

**Affiliations:** 1 School of Social Work, University of Maryland, 525 West Redwood Street, Baltimore, MD 21201, USA; 2 College of Public Health, University of Kentucky, 151 Washington Ave., Lexington, KY 40506, USA; E-Mails: jess.clouser@uky.edu (J.M.C.); swest1@email.uky.edu (S.C.W.); mary.webster@uky.edu (M.W.M.); 3 College of Nursing, University of Kentucky, 315 College of Nursing Bldg., Lexington, KY 40506, USA; E-Mail: dbreed01@email.uky.edu

**Keywords:** worker injury, occupational health, Latino workers, agriculture, occupational safety, immigrant workers, horse-related injuries

## Abstract

Animal production is a dangerous industry and increasingly reliant on a Latino workforce. Within animal production, little is known about the risks or the occupational hazards of working on farms involved in various aspects of thoroughbred horse breeding. Extant research suggests that horse workers are at risk of musculoskeletal and respiratory symptoms, kicks, and other injuries. However, limited known research has examined the experiences of the industry’s workers, including immigrant workers, despite their prominence and increased vulnerability. Using data collected from thoroughbred farm representatives via a phone-administered survey, a 2-hour face-to-face semi-structured interview, and farm injury logs, this article identifies and describes types of injuries experienced by workers (N = 284) and their surrounding circumstances. Results indicate that general injuries and musculoskeletal strains, sprains, and tears account for a majority of injuries among workers on thoroughbred farms. Upper limbs and extremities are most frequently injured, while direct contact with the horse accounted for over half of all injuries. No differences in the diagnoses or distribution of injury were found by ethnicity; however, Latinos were more often struck by or trampled by a horse while non-Latinos were more often injured by an insect or plant. Implications and opportunities for future research are discussed.

## 1. Introduction

Horses play a large role in many USA states’ agricultural economies. Nationwide, the horse industry contributes over $39 billion to the USA economy and employs 460,000 full-time workers in various roles [1]. Horses perform a variety of functions, ranging from recreation and racing to assisting in farm and ranch work. Numerous studies have documented the dangers inherent in riding horses for pleasure or sport [2,3,4], with recent studies highlighting the hazards particular to racetracks for their demanding and hazardous work conditions [5]. Less attention has been paid to the farms where horses are bred, raised, boarded, and sold. 

Horse breeding, as a part of animal production, is a dangerous subset of a hazardous industry: agriculture. In fact, in 2011, animal production experienced an incidence rate of 6.4 non-fatal injuries per 100 full-time workers compared to 5.1 per 100 workers employed in crop-related industries, and 3.6 among all industries [6]. As such, horse breeding operations likely harbor many of the injury hazards that other large animal operations face: the threats of kicks, falls and tramplings [7,8], potential exposure to toxic medicines [9,10], animal bites [8,11,12], and high postural loads when bending or twisting [13]. These are in addition to hazards documented among equestrian sports and racing facilities: repetitive chronic pulling on upper extremity joints [14] and potential for head, face, neck chest, abdomen, and upper extremity injuries [5,11]. 

Another gap in our understanding about injury risks on horse farms is the characteristics of workers employed on them. Literature documenting “the backstretch,” or the stables located at racetracks, notes that these workers, chiefly grooms, are largely Latino, and often from Mexico or Guatemala [15]. Anecdotal evidence suggests this is also true on thoroughbred farms. Evidence indicates that Latinos experience poorer occupational health outcomes than all other workers in the USA across all industries [16,17]. For example, in 2004, the occupational fatality rate among Latinos was 5.0 per 100,000 *vs.* 4.1 per 100,000 for non-Hispanic whites [18]. While the total number of nonfatal injuries has steadily declined since 2000, the proportion of minorities represented in total nonfatal injuries has steadily increased with Latinos comprising more than half the minority cases [19]. 

Looking specifically at agriculture, fatality and injury rates among Latinos are seven times the national average [20] and agriculture is one of the top four industries with the highest fatality rates among Latino/Hispanic workers [21]. Lower educational attainment, limited English language proficiency, inadequate access to safety equipment, limited or no training, and economic pressures are possible reasons for higher injury rates among Latino workers [17,22,23,24,25].

Although an expanding body of research on the health and safety of Latino farmworkers has been conducted within several sectors of agriculture [26,27,28], there has been limited attention to workers in the equine sector. One small study of occupational health and injuries among Latino horse workers found a high prevalence of physical demands, musculoskeletal discomfort and injuries [10]. Such injuries may be related to the varied tasks horse workers perform, including animal-related tasks such as brushing, bathing, feeding, walking, and riding horses, or to other manual labor such as mucking stalls, mowing, weed eating, and working with machinery [29]. Given the lack of research on the occupational health of workers employed in the equine industry, including documentation of injury trends by ethnicity/race, we do not know if Latinos are at higher risk of injury than their non-Latino counterparts, or whether they are more prone to certain types of injuries.

Similarly, little research about hazards associated with working with animals has focused on horse breeding farms, particularly thoroughbred operations that require live breeding and are host to particularly athletic, temperamental animals. Given that thoroughbred racing is a prominent equestrian sport with thoroughbred breeding operations supporting it in the United States and increasingly in the world [11,30], specific attention to breeding operations’ hazards and the injuries experienced on these farms is merited.

This study aims to the address the gap in knowledge by describing the demographic details of frontline workers employed on thoroughbred farms and the type and nature of injuries experienced by workers on thoroughbred farms, including assessing differences in injuries experienced by Latino and non-Latino workers and the circumstances surrounding these injuries. 

## 2. Methods

### 2.1. Overview

The data for this study were collected as part of a larger community and employer-engaged research project titled: *Thoroughbred Worker Safety and Health Study* or *Salud y Seguridad del Trabajador Equino*. The study was designed to systematically document the demographics, working conditions, and occupational health of Latino farmworkers employed on thoroughbred horse farms in the southeastern United States, and to develop intervention materials to promote the safety and health of these workers. The study engages members of the Latino community and the thoroughbred industry through two advisory groups: the *Community Advisory Council* and the *Industry Advisory Council*. This article uses data from the first phase of the study which collected information about injuries from injury logs and in-depth interviews with farm managers. Phase two of this study will gather detailed information from Latino workers about injuries they have experienced. Collecting information about injuries from multiple sources via mixed-method approaches will offer a comprehensive look at injury trends on horse farms and provide rich information from which safety and health interventions will be designed. 

### 2.2. Eligibility, Sampling, and Recruitment

Farms were eligible to participate in phase one of this study if they: (1) were engaged in breeding or boarding thoroughbred horses, (2) employed at least one Latino worker, and (3) were located in the southeastern United States. Inclusion criteria for the employer representative required that he/she be 18 years of age or older and hold a position of farm owner, manager, or personnel administrator (e.g., Human Resources Manager) at the recruited farm. Data were collected between October 2012 and March 2013. 

A sampling frame of 82 thoroughbred farms was developed using names of farms identified by the Industry Advisory Council. It is estimated that approximately 70% of thoroughbred farms in the designated region employ less than 10 workers (small farms), 15% employ 11–25 workers (medium farms), and 11% employ more than 25 workers (large farms) [31]. Because there is no known database that reports horse farm size by number of employees, farm size was estimated by advisory council members according to the above definitions. A sampling frame was populated to match the distribution of small, medium, and large farms in the region. Farm size was confirmed or corrected as part of the interview process.

Due to the time required to participate, the sensitivity of the topic, and the close-knit nature of the industry, it was strongly advised by the industry advisory council that convenience sampling be used instead of randomly sampling thoroughbred farms. Without an introduction to the study from a respected member of the thoroughbred community, entry onto the farms would be very difficult. Thus, a letter, written and signed by two members of the Industry Advisory Council and the Principal Investigator was sent to the contact person at the farm describing the study’s goals and methods. Within a week of sending the letter, a trained interviewer contacted the farm by phone. If the owner/manager agreed to participate, the interviewer established eligibility and enrolled them in the study.

Eighty-two farms received the initial letter inviting their participation in the study. Twenty (24.4%) were ineligible either because they did not fit the study’s criteria, were no longer in operation, or had disconnected telephone lines. Of the remaining 62 farms, 32 completed the phone interview, for a response rate of 51% and 26 farms participated in both the phone interview and the face-to-face in-depth interview for a 41% participation rate. Among these 26 farms, one farm reported that no injuries had occurred on the farm in the previous five years, one farm declined to provide injury data, and two farms, though initially agreeing to provide injury logs, failed to provide injury documentation. Thus, the analysis presented in this paper uses data collected from 22 farms that provided detailed information about recent injuries, or 36% of the original sampling frame. Participants were given a framed certificate and a study-branded water bottle for their participation. Research procedures were approved by University of Kentucky’s Non-Medical Institutional Review Board.

### 2.3. Data Collection

Three main types of data collection strategies were used to gather information about injuries on thoroughbred farms: a phone interview, an in-depth, semi-structured, face-to-face interview, and the collection of de-identified injury logs or other self-collected data from farms. The content of the 20-minute telephone interview included seventy-three questions about farm and workforce demographics, organizational practices, and occupational safety. The in-depth semi-structured interview gathered information about job tasks, work organization, occupational hazards, safety climate, and injuries. Interviewers probed for additional information about recent injuries or illnesses to collect more detailed information about the context of the injury events. The in-depth interview was digitally recorded and later transcribed for analysis. The third data collection strategy entailed collecting copies of de-identified workers’ compensation claims, Occupational Safety and Health Administration (OSHA) reporting logs, and internal farm injury logs. Overall, the distribution of sources for injury information was: OSHA logs (49.3%), workers’ compensation reports (21.5%), informal tracking sheets (11.3%), workers’ compensation summary forms (9.5%), and the in-depth interview (8.5%). Further information was captured from qualitative interviews for 84.2% of the injury incidents where data was missing from the primary source. Representatives of 22 farms provided usable information about worker injuries that occurred on their farms over the past five years, yielding a total of 284 unique injury incidents.

### 2.4. Measures

The outcome measure for this analysis was worker injuries that occurred on the farm in the last five years. Worker injuries are those injuries that were reported by a worker to a farm representative, recorded in one of the previously identified injury logs or witnessed by a farm representative. Detailed information about each worker injury was collected from injury logs. If no such documentation was submitted by a participating farm, injury incidents were collected from that portion of the in-depth interview and coded accordingly. Each incident was coded for the: (1) *diagnosis* or type of injury (bruise, fracture, *etc.*); (2) *distribution* or location of the injury (head, neck, *etc.*); and (3) *mechanism* or action that led to the injury (*i.e.*, kick, fall, *etc.*). “Diagnoses” were often, but not necessarily, clinically diagnosed. In cases where multiple body parts were involved, each diagnosis and injury site (distribution) was coded separately, whereas, each injury event was assigned one mechanism. Injury incidents were coded for sex of injured worker, source of injury (horse or non-horse), and ethnicity, which was dichotomized into Latino and non-Latino. Horse-related injuries were defined as injuries resulting from tasks directly involving a horse (e.g., riding, brushing, or leading), whereas non-horse related injuries were defined as all other injuries in which directly handling a horse was not involved (e.g., fall from structure). Because demographic characteristics (*i.e*., sex, ethnicity) are not standard fields on OSHA or workers’ compensation claim reports, this information was either gathered or confirmed during interviews with farm representatives or extrapolated from available descriptors in the logs. However, some injury incidents have missing data. Of 284 injury incidents, 37 lack information about sex and 22 lack information about ethnicity. Similarly, six injuries lacked enough information to qualify whether it was a horse or non-horse related injury. In light of missing data, our analysis and subsequent results report information on all 284 injury incidents whenever possible, because the data available provides meaningful insights into the range and context of injuries experienced by horse workers. 

### 2.5. Analysis

Quantitative data collected from phone surveys were entered into SPSS (Version 21, IBM Corp Armonk, NY, USA), while information collected from secondary research documents was entered into an Excel^TM^ (Microsoft, Redmond, WA, USA) database. Both files were exported into SAS (Version 9.3, SAS Institute, Cary, NC, USA) [32] for analysis. Transcripts from the in-depth interviews were entered in Atlas-ti (Version 6, Atlas.ti Scientific Software Development GmbH, Berlin, Germany) [33] and coded by three research team members to gather information about injury incidents. The coding scheme followed that of the secondary documents: the mechanism, distribution and diagnosis of the injury incident were identified and coded, as were the gender and the race/ethnicity of the injured worker. Injury incidents and coded descriptors for farms that did not provide injury logs were entered into the Excel^TM^ database and included in the quantitative analysis. When farms provided injury logs, data gleaned from the qualitative interviews were cross-referenced to assure accuracy. 

The goal of our analysis was to identify the injuries experienced by workers on thoroughbred farms as well as the context surrounding those injuries. Thus, univariate descriptive statistics, including frequency, mean, and median calculations, were performed to assess the basic demographic profile of the frontline farmworkers on the 22 farms as well as the injury profile of the workers in the injury log. Bivariate analysis, chi-square, and Fisher’s exact tests were conducted to explore relationships between injury distribution, mechanism and diagnosis, and ethnicity. Injuries were grouped into those that were horse-related and others that were not. 

## 3. Results

### 3.1. Farm and Worker Characteristics

The median number of acres devoted to thoroughbred operations across the 22 farms in the sample was 378 acres with a mean of 153 thoroughbred horses per farm (See Table 1). Farm size was relatively evenly dispersed, with a little over a third of farms in both small and medium categories (36.4%), and under a third being large farms (27.3%). However, the average number of workers on each farm varied widely from 1 to 230. As detailed in Table 1, a large majority of farms in the sample engaged in four main functions: horse sales—selling horses on behalf of oneself or others (100%), breeding—being engaged in mating or foaling (95.5%), boarding—caring for and possibly foaling horses that one does not own (77.3%), and racing—racing horses in competition (72.7%). In addition to their work with thoroughbreds, over a third of farms (36.4%) raised other commodities, chiefly crops (e.g., hay and corn) and livestock (e.g., cattle) (see Table 1). 

Overall, farms employed an average of 34 (SD = 52.6) year round workers in a variety of positions (e.g., administrative staff, managers, grooms). Looking specifically at frontline workers, or farmworkers, the mean was 26 (SD = 40.6), with a median of 11 (see Table 1). Most farms employed seasonal (63.6%) and contract workers (72.7%) in the past year, while just under half (45.5%) employed part-time employees (see Table 1). Contract labor was most often used for riding or breaking (early training) horses, working horse sales, landscape/maintenance tasks, and farm renovations. 

The most commonly held farmworker positions were grooms, maintenance workers, and night watchmen. According to interviews conducted with farms, grooms are generally assigned to work with horses and are responsible for spreading and cleaning bedding in stalls, feeding, brushing, bathing, and administering medicines to horses, as well as turning them out to pasture. Maintenance workers are responsible for the day-to-day upkeep and repair of the farm, including operating farm equipment. Night watch workers work the night shift, primarily during foaling season (January–June), to monitor mares about to give birth. Some farms hire “general farm hands,” or workers who are often responsible for many or all of the tasks of grooms, maintenance workers, and night watchmen.

**Table 1:** Farm and employment characteristics (N = 22).

		Mean	SD	Range	Median	IQR
Acres devoted to thoroughbred operation		875	1,294.8	30–6000	387	894
No. of thoroughbreds		153.1	132.5	6–516	100.5	127
No. of all workers on farm		34	52.6	1–230	13	22
No. year round farm workers (frontline)		25.8	40.6	1–180	11	13
		**N**	**%**			
Farm size by No. year round employees						
	Small (1–10 workers)	8	36.4			
	Medium (11–25 workers)	8	36.4			
	Large (>25 workers)	6	27.3			
Thoroughbred operation includes ^1^						
	Sales	22	100.0			
	Breeding	21	95.5			
	Boarding	17	77.3			
	Racing	16	72.7			
Also raise other crops/commodities^ 1^		8	36.4			
	Crops	6	27.3			
	Livestock	4	18.18			
No. farms with PT year round employees		10	45.5			
No. farms with seasonal employees		14	63.6			
No. farms with contract employees		16	72.7			
		**N**	**%**			
Most common jobs on the farm ^1, 2^						
	Grooms	22	100.0			
	Maintenance worker	17	77.3			
	Night watch	16	72.7			
	Landscapers	11	50.0			
	Exercise Rider	8	36.4			
	General Farm Hand	4	18.2			
No. of farms where workers perform multiple job tasks		14	63.6			

Notes:^ 1 ^Percentages do not equal 100 because responses are not mutually exclusive; ^2^ Farms were asked to list their top three jobs for number of workers employed.

Over 80% of the aggregated farmworker labor force across all 22 farms was men (Table 2). Latinos comprised the majority (61.8%) of each farm’s year round, frontline farmworkers (data not shown), while overall comprising about half (49.8%) of the aggregated frontline labor force. Non-Latino blacks comprised a small portion of the aggregated frontline workforce (3.5%). Just under half of the frontline labor force of farmworkers were foreign-born (43.7%), or had a native language other than English (44.2%) (see Table 2). Ninety-five percent of farms employed workers from Mexico (data not shown), though across all farms, 25 countries were represented by the foreign-born labor force. According to farm representatives, the languages spoken by foreign-born workers were mostly Spanish, English, and Portuguese (data not shown). 

**Table 2:** Estimated characteristics of farmworkers (frontline workers) aggregated across 22 farms (N = 568).

Demographic Characteristics		N	%
Sex			
	Male	476	83.8
Race			
	Latino	283	49.8
	White (non-Latino)	265	46.7
	Black (non-Latino)	20	3.5
Nativity			
	Born in country other than USA	248	43.7
Language			
	Native language not English	251	44.2

### 3.2. Injuries

A total of 284 injuries were documented. A large majority of the reported injuries were experienced by men (81.4%) and over half (57.6%) were experienced by non-Latinos (data not shown). Among all injuries, 30 (10.6%) occurred on small farms, 25 (8.8%) occurred on medium farms, and 229 (80.6%) occurred on large farms (data not shown). Horses were more frequently the direct source of injuries compared to non-horse related events, comprising 56.8% of the documented injuries. 

The most prevalent diagnosis (see Table 3) was a general injury category, comprising nearly a third of the total (29.2%). This category was created when a specific diagnosis was not determined, but general “pain” or “hurt” was described (e.g., “kicked by yearling”, “injured right middle finger”, “kicked in right temple” or “injured right thumb and wrist, fell back”). This was closely followed in prevalence by musculoskeletal strains, sprains, and tears (28.2%), followed by contusions (13.4%), and broken or crushed bones (10.9%). No difference was found between Latino and non-Latino workers in the type of injury diagnosis. However, differences were found between horse and non-horse related injuries for five diagnoses. Horse-related tasks were more often associated with general injuries (*p* = 0.000), and contusions (*p* = 0.033), whereas non-horse related tasks were more often associated with musculoskeletal sprains, strains, and tears (*p* = 0.001), irritations (*p* = 0.033), and stings (*p* = 0.002). 

Distribution of injuries was clustered into 13 regions of the body (see Table 4). Overall, upper extremities accounted for the highest number of injuries: specifically, wrists, fingers, and hands (17.8%), and arm and shoulders (17.0%). Ankles, feet, or toes were close behind (14.4%), as were injuries to the back or spine (13.3%). No statistically significant differences were found between Latino and non-Latino workers in the location of the injury. However, differences were found between horse and non-horse injuries in four sites. Horse-related tasks were more often associated with head (*p* = 0.018), and chest (*p* = 0.007) injuries, while non-horse tasks were more often associated with back and spine (*p* = 0.001), knee (*p* = 0.029), and neck injuries (*p* = 0.001). 

**Table 3:** Diagnosis of injury in cases: total injuries and designation as horse and non-horse related and Latino and non-Latino demographic.

Diagnosis of Injury	Number of Diagnoses	Number	Number	Number	Number
		Diagnoses, Horse-related (%)	Diagnoses, Non-horse Related (%)	Diagnoses, Latino (%)	Diagnoses, Non-Latino (%)
	(N = 287) ^1^	(N = 278) ^2^		(N = 262) ^4^	
General injuries	83 (29.3)	60 (73.4)	21 (26.6) ***	35 (43.2)	46 (56.8)
Strains, sprains or tears	80 (28.2)	33 (41.8)	46 (58.2) ***	31 (40.8)	45 (59.2)
Contusions	38 (13.4)	27 (73.0)	10 (27.0) *	14 (50.0)	14 (50.0)
Broken/crushed bones	31 (10.9)	22 (69.0)	9 (31.0)	15 (53.6)	13 (46.4)
Cuts	26 (9.2)	10 (40.0)	15 (60.0) ^˄^	9 (36.0)	16 (64.0)
Irritation ^3^	10 (3.5)	2 (22.2)	7 (77.8) *	3 (33.3)	6 (66.7)
Stings	7 (2.5)	0 (0.0)	7 (100.0) **	1 (14.3)	6 (85.7)
Inflammation	5 (1.8)	4 (80.0)	1 (20.0)	3 (60.0)	2 (40.0)
Joint dislocation	4 (1.4)	1 (25.0)	3 (75.0)	2 (66.7)	1 (33.3)
Other diagnoses	3 (1.1)	1 (33.3)	2 (66.7)	1 (33.3)	2 (66.7)

Notes: * *p* < 0.05, ** *p* < 0.01, *** *p* < 0.001, ^˄ ^*p* < 0.08, approaching significance regarding horse *vs.* non-horse related incident; ^1 ^Events with multiple diagnoses were coded for each diagnosis obtained; ^2 ^Six cases had missing data for horse/non-horse related; ^3 ^Irritation chiefly comprised of skin or eye irritation; ^4^ Twenty two cases had missing data for ethnicity.

**Table 4:** Distribution of injury in cases: total injuries and designation as horse and non-horse related and Latino and non-Latino demographic.

Site of Injury ^1^	Number of Sites	Number Sites, Horse-related (%)	Number Sites,	Number Sites, Latino (%)	Number Sites,
	(%)		Non-horse Related (%)		Non-Latino (%)
	(N = 271)^ 2^	(N = 265) ^3^		(N = 252) ^4^	
Wrist, fingers or hands	49 (17.8)	26 (55.6)	20 (44.4)	16 (35.6)	29 (64.4)
Arms and shoulder	46 (17.0)	31 (67.4)	15 (32.6)	17 (38.6)	27 (61.4)
Ankles, foot or toes	39 (14.4)	22 (57.9)	16 (42.1)	19 (51.4)	18 (48.6)
Back or spine	36 (13.3)	12 (33. 3)	24 (66.7) ***	12 (33.3)	24 (66.7)
Face	27 (10.0)	18 (72.0)	7 (28.0)	13 (52.0)	12 (48.0)
Head	22 (8.2)	18 (81.8)	4 (18.2) **	9 (45.0)	11 (55.0)
Knee	20 (7.4)	7 (35.0)	13 (65.0) *	7 (43.8)	9 (56.3)
Chest	14 (5.2)	13 (92.9)	1 (7.1) **	5 (35.7)	9 (64.3)
Leg	12 (4.4)	8 (66.7)	4 (33.3)	5 (55.6)	4 (44.4)
Abdomen	9 (3.3)	5 (62.5)	3 (37.5)	3 (37.5)	5 (62.5)
Groin	8 (3.0)	4 (50.0)	4 (50.0)	4 (57.1)	3 (42.9)
Hip or pelvis	6 (2.2)	2 (33.3)	4 (66.7)	3 (50.0)	3 (50.0)
Neck	5 (1.1)	0 (0.0)	5 (100.0) **	3 (60.0)	2 (40.0)

Notes: * *p* < 0.05, ** *p* < 0.01, *** *p* < 0.001 regarding horse *vs.* non-horse related incident; ^1^ Events involving multiple body parts were coded for each effected part; ^2 ^Thirteen cases had missing data for site; ^3 ^Nineteen cases had missing data for site and/or horse/non-horse related; ^4^ Thirty two cases had missing data for ethnicity and/or site.

There were 16 principal mechanisms of injury, which were separated into horse and non-horse related (see Table 5). The top two mechanisms were both horse-related: kicks (19.8%) and struck by horse—*i.e.*, being hit by a horse with something other than a hoof (11.5%).

The fourth most prevalent mechanism, trampled/stepped on (8.6%), was also horse-related. Contact with equipment/tools (8.3%) was the most prevalent non-horse related mechanism. Differences were found between Latino and non-Latino workers for three individual mechanisms. Latino workers were more likely to be struck by (*p* = 0.004) and trampled or stepped on (*p* = 0.003) by a horse, while non-Latino workers were more likely to have had an injury related to insects or plants (*p* = 0.02).

**Table 5:** Mechanism of injury overall and by Latino and non-Latino demographic.

Injury Mechanism	Number of Mechanisms (%)	Number of Mechanisms, Latino (%)	Number Mechanisms, Non-Latino (%)
	(N = 278) ^1^	(N = 262) ^2^	
**Horse-related Injuries**			
Kick	55 (19.8)	24 (48.9)	25 (51.2)
Struck by	32 (11.5)	20 (66.7)	10 (33.3) **
Trampled/stepped on	24 (8.6)	16 (72.7)	6 (27.3) **
Horse jerked or pulled	19 (6.8)	8 (42.1)	11 (57.9)
Horse related overexertion	8 (2.9)	4 (50.0)	4 (50.0)
Fall from horse	7 (2.5)	1 (16.7)	5 (83.3)
Contact with horse related equipment	6 (2.2)	1 (20.0)	4 (80.0)
Bite	5 (1.8)	1 (20.0)	4 (80.0)
Other horse related	2 (0.7)	0 (0.0)	2 (100)
**Total **	**158 (56.8)**	**75 (51.4)**	**71 (48.6)**
**Non-horse Related Injuries**			
Contact with equipment or tool	23 (8.3)	6 (28.6)	15 (71.4)
Lifting	19 (6.8)	6 (33.3)	12 (66.7)
Slip/trip/fall on ground	19 (6.8)	4 (26.7)	11 (73.3)
Fall from equipment or structure	15 (5.4)	5 (38.5)	8 (61.5)
Struck by non-horse object	11 (4.0)	3 (27.3)	8 (72.7)
Insect/plants	11 (4.0)	1 (9.1)	10 (90.9) *
Overexertion	8 (2.9)	2 (25.0)	6 (75.0)
Contact with foreign object	10 (3.6)	4 (44.4)	5 (55.6)
Other non-horse related	7 (2.5)	2 (28.6)	5 (71.4)
**Total**	**120 (43.2)**	**32 (29.1)**	**78 (70.9)**

Notes: * *p* < 0.05, ** *p* < 0 .01; ^1 ^Six cases had missing data for mechanism; ^2 ^Twenty two cases had missing ethnicity data.

## 4. Discussion

### 4.1. Findings

Our study of 22 thoroughbred horse farms and the injury incidents reported by workers to managers on these farms reveals three main findings: (1) it describes the nature of injuries that occur on thoroughbred farms, (2) it reports on the relative frequency of horse-related injuries compared to non-horse related injuries, and (3) it provides information about the demographic composition of farmworkers and injured farmworkers who reported injuries. Although our research design collected more detailed information about worker demographics and events leading up to the injury than is included national injury surveillance systems—including information about ethnicity—it was still limited by the fact that workers must have reported an injury to a manager, supervisor, or other farm representative in order to have been included in our analysis. Thus, we are careful to note that these injuries may not reflect the full spectrum of every injury experienced on these farms. Yet, these findings are important because scant literature exists on injuries to workers on horse farms. As such, this is one of the first studies to describe patterns of injuries associated with handling horses known for their strength, quick responses, and high-strung temperaments. It is also one of the first studies to offer details about a previously understudied and vulnerable workforce in the equine sector of the agricultural industry. 

Our first finding relates to the diagnosis and distribution of injuries reported on thoroughbred farms. Nearly a third of the injuries fell into the most common category, “general injuries”, a term that was ascribed to injuries without a specific diagnosis. Because these injuries did not include a diagnosis they may be less severe than injuries that were documented by a health care provider (e.g., “employee was holding a foal’s back leg while brushing and foal kicked employee in forehead”). If one adopts this interpretation, these findings indicate that minor injuries may be relatively common on horse farms, especially among individuals working directly with horses. This may explain the discrepancy with findings from a Japanese study of thoroughbred worker injuries obtained from hospital records which showed a preponderance of contusions, fractures, and abrasion/laceration as the most dominant injuries sustained [11]. Because hospitalization was required for inclusion in that study, minor injuries would not have been included. However, it is also possible that these injuries simply may not have been described in sufficient detail in the injury logs, which argues for making the case to farm representatives to include more detail in injury reporting to enable researchers a better ability to determine causation and make helpful recommendations to improve farm and worker outcomes. 

After “general injuries”, the most common injury diagnosis was musculoskeletal sprains, strains, and tears. The prevalence of these injuries reveals the strenuous nature of the work, which is often comprised of bending, twisting, and throwing while mucking or sweeping stalls. These tasks, and the particularly high postural load inherent in stable work, have been documented by Swedish riding instructors and stable hands [13,14], among whom mucking (removal of manure) and handling straw or hay were responsible for the greatest work load. 

Taken together, upper limbs and extremities were most frequently injured at the farms in our sample, followed by ankles, feet, and toes. The frequency of upper limb and extremity injuries is consistent with musculoskeletal discomfort in riding instructors in Sweden [13] and a NIOSH review of injuries documented in the thoroughbred and standard-bred industries in the USA [5]. Both upper and lower limb and extremity injuries are consistent with hospitalization records of thoroughbred workers in Japan [11] and “sports related injury riding” patients in the U.K. [34]. This finding lends support to the idea that the nature of injuries sustained by horse workers may be different than those experienced by those riding for sport or pleasure, in which head injuries have historically been more prevalent [4,35]. It also supports the argument that severe injury prevalence among riders is shifting from the head to upper and lower extremities [34], perhaps due to the increased use of helmets among riders. Moss *et al.*’s [34] recommendation to develop and promote protective gear for the upper and lower extremities, particularly the wrist, may prove fruitful for horse farmworkers.

Despite the higher frequency of injuries in the upper and lower limbs in general, in our sample, head and chest injuries were more frequently experienced by workers performing horse-related tasks compared to tasks not directly involving a horse. Given the potentially critical nature of injuries to the head or chest, strategies to prevent these injuries by protecting these areas remain important. 

Our second finding focuses on the mechanism of injuries and the relationships that horse-related tasks and non-horse related tasks have with injuries on these farms. General injuries, contusions, and injuries to the head and chest more often happened when working with horses, while non-horse related tasks were more often associated with injuries to the back and spine, knee, and neck as well as musculoskeletal sprains, strains and tears; irritation; and stings. These findings show that while the horse accounts for the greatest threat, there are many facets of working on a horse farm that carry risk of injury.

Kicks were the most common mechanism of injury among our sample, consistent with injuries incurred by Japanese thoroughbred workers [11]. When combined with being struck by, trampled, bitten, jerked, or falling from the animal, horses themselves accounted for half of all injuries. This is not surprising, the horse is a frequently unpredictable animal whose well developed fight or flight instinct may cause it to react to even small changes in the environment [29,36]. In fact, several researchers have concluded that increased exposure to horses was more influential than experience regarding one’s risk of injury [3,35]. Our finding that the animal provides the greatest source of injuries on the farm is consistent with a review of workers’ compensation claims related to livestock operations in Colorado [8]; however, the proportion of injuries attributed to the horse in our sample (50.0%) was even higher than the 31% attributed to livestock handling in that study. 

It should be noted that nearly half of all injuries on the farm involved mechanisms and tasks that don’t directly involve the animal, such as mucking stalls; performing farm maintenance or landscaping with machinery, equipment, or tools; or lifting heavy loads. This is an important distinction to make on horse farms due to the wide variety of tasks to which workers may be exposed. Depending on the size and organization of the farm, these tasks could be delegated to different worker groups (e.g., grooms *vs.* maintenance) or to the same worker (e.g., general farm hand). 

Our third finding pertains to the demographic information this study provides relative to employees’ injury experiences on horse farms. Similar to farms in other agricultural sectors [37,38,39], farms in this study’s sample heavily depend on Latino workers, who held half of all farmworker positions in the sample. The injuries reported, however, were not equally divided between Latinos and non-Latinos. There are several possible reasons for this. First, the information on worker injuries is inclusive of all workers on the farms—frontline workers as well as supervisors and managers. Reports from farm representatives indicate that managers and supervisors on thoroughbred horse farms are more likely to be non-Latino, and because of their leadership role on the farm may be more likely than frontline workers to report an injury when it occurred. Second, Latino workers may have underreported injuries to their supervisor. Research conducted with vulnerable worker groups, like immigrant farmworkers who may fear retribution or termination if injured, indicates that they may be reluctant to report injuries to supervisors [29,40]. A study of injuries among Latino farm workers, from the worker perspective, revealed that one out four had been injured in the last year [24]. Such findings expose limitations in surveillance mechanisms that rely on workers reporting injuries to a manager for them to be counted [41]. Consequently, a worker may be a more reliable source of information about employee health than a farm representative, and that information is not available in this analysis. 

In light of these limitations, we found no differences in the specific diagnoses or distribution of the injuries reported by ethnicity. This suggests the type and location of the injuries may be similar for all workers, regardless of ethnicity. In contrast, differences in the specific mechanism of the injuries were found between Latino and non-Latino workers. Latinos were more likely to report being struck by or trampled by a horse than non-Latinos, while non-Latino workers were more likely to report injuries involving insects or plants. This former finding may be attributed to an increased prevalence of Latino workers in jobs directly related to working with horses or indicative of Latinos not receiving adequate training that may reduce trampling or struck-by incidents. It’s also possible that Latinos may be more likely to report more severe events (e.g., trampled by a horse), than seemingly “minor” events (e.g., insect or plant-related incident). Future research is needed to better understand the nuances surrounding injuries experienced by Latino and non-Latino farmworkers, and to better understand what factors may contribute to differences between the two worker populations. 

### 4.2. Limitations

This exploratory study is part of a larger effort to delineate the inherent hazards, injuries, and work organization factors on horse farms, which are chief employers of a non-English speaking workforce. Its mixed-methods research design, which incorporates both qualitative and quantitative data collection in addition to formal and informal injury logs, helps to address inherent difficulties in standard injury surveillance programs, including the lack of contextual information about events leading to injuries, limited demographic information about workers, and the omission of small employers from reporting requirements. 

However, it is also limited by its design in the following ways. First, we cannot presume that our small, nonrandom sample is representative of all horse farms, as participating farms may have been more motivated to participate because they have strong health and safety programs in place. Also, although 32 farms participated in the survey, only 22 were included in the final analysis due to their providing information about recent injuries or illnesses. Some farms did not provide data because they reported that no injuries were experienced in the past five years. Other farms did not keep records or declined to share this information. As such, the injury information garnered from farm records and conversations may be inherently biased. Further, despite our attempt to mimic the size distribution of the industry, which is predominantly composed of small farms, the reality is that small farms are often run by a single or small staff of manager/owners who do not have much time to spare. Although we contacted a greater number of small farms, a greater percentage of medium and larger farms consented. 

Another limitation is that exposure of different worker groups is not assessed in this analysis, though we learned that the positions and schedules vary according to responsibility. Animals need care and feeding every day of the week, every month of the year. Consequently, the workers in charge of caring for horses work long weeks, with a six-day workweek being an industry standard for grooms. This schedule is intense, with little time away from work to recover, and significant exposure to the animals. Evidence from a small study of horse and crop workers suggests that increased work hours increases the odds of work-related injury [24]. Further information about the work environment at the farm and the experiences of the workers should be gathered in order to assess the relationship between work schedules, exposure, and injury risk. 

It should be noted that the injuries reported in this study do not necessarily reflect all injuries that occurred on the farms in the sample over a five-year period. Some farms in our sample provided information about injuries that occurred in the past five years; other farms only included injuries that had occurred in the past year, or verbally described recent injuries experienced. Consequently, these injuries are illustrative of the types of injuries that may occur on farms and the demographic profile of the workers they impact. 

Through the mixed-methods research design, and the collection and triangulation of multiple types of data, an effort was made to minimize the limitations associated with national surveillance mechanisms. Despite these efforts, standardized injury logs were a primary source of data for many of the injuries in the analysis and are limited in similar ways, including: (1) the reliance on a manager’s awareness of an injury for it to have been recorded, (2) the absence of demographic data such as age or ethnicity on many standardized injury logs. Collecting data from multiple sources enabled many of these limitations to be overcome; however, missing data remained in many of these fields, which should be considered when interpreting the analysis. Even with these limitations, because so little is known about the work tasks, hazards and injuries experienced by horse workers, this descriptive information is important for determining priorities for future research.

## 5. Conclusions

Results from this research indicate that thoroughbred horse breeding farms involve a variety of tasks that put workers at risk of both acute and chronic injury. The risk of being kicked by or struck by a horse is always present, as is the risk of straining muscles or ligaments through the strenuous work associated with stable tasks and other farm duties. In fact, direct contact with the horse accounted for over half of all injuries. Our results indicate that Latinos comprise a substantial part of the frontline labor force on thoroughbred farms in this study and that the diagnosis and distribution of the reported injuries are similar among Latinos and non-Latinos. However Latino workers were more likely to be struck by or trampled by a horse than non-Latinos. Future research is necessary to identify injuries experienced by Latino and non-Latino workers from their point of view, to better understand the reporting patterns of Latino and non-Latino workers, and to identify the organizational factors that may increase risk of injury among all farm workers.
